# Policy agendas of the American state legislatures

**DOI:** 10.1038/s41597-025-05621-5

**Published:** 2025-07-22

**Authors:** Ethan Dee, Alex Garlick

**Affiliations:** 1 Independent Researcher, Received Ph.D at the University of Illinois at Urbana-Champaign, Champaign, Illinois USA; 2 University of Vermont, Political Science Department, Burlington, Vermont USA

**Keywords:** Government, Politics, Law, History

## Abstract

State legislatures in the United States handle a number of important policy issues, but pose a challenge for researchers to observe because they are not organized by any central agency. We use a machine learning model based on the “transformer” architecture and contextual word-piece embeddings to code the universe of bills introduced in the states since 2009 (about 1.36 million bills) into 28 policy areas. Validation exercises show our method compares favorably with hand-coded estimates of bill policy areas while offering far greater coverage than legacy human-supervised “dictionary” methods. We explain how researchers can use these estimates to investigate sub-national governance in the United States.

## Background & Summary

A legislative agenda reveals what a government is working on at the moment, and can provide a historical index of the priorities of a legislature over time. That is, if it is properly organized. The US Congress has been organized for public consumption by the Library of Congress (via congress.gov) and researchers^1^ for many years, leading to reams of scholastic work on the body. However, there is no such analog at the state level, despite the fact that state legislatures regulate sizable markets and handle consequential matters from election law to civil rights. Non-government organizations and private firms have been digitizing state legislative histories for a number of years, but they have not been systematically coded.

This paper describes a machine learning method to code the universe of state legislation by policy area since about 2009, and provides its output. Significant advances in computational hardware have made it possible to use machine learning models to approach the reliability of hand-coders at a fraction of the cost. Using our estimates researchers can track the levels and changes of bills introduced in 28 policy areas, from broad (macroeconomic policy) to specific (public lands and water management). Temporal change produces important insights, for example, the most common policy area in the states is health care. State legislators increased a spate of bills on the topic following major federal action (the 2010 Affordable Care Act), but there was no such spike following the failed attempt to “repeal and replace” the Affordable Care Act in 2017.

Previous scholars have used a list of keywords that capture the essence of topics to measure pockets of the agenda, such as “health” being a keyword for the “Health” topic, or “highways,” “transit,” and “airports” mapping to “Transportation.” But there are limitations to dictionary-based approaches such as this, or other “bag of words” models based solely on the prevalence of words. For example, Garlick (2023)^2^ used a legacy dictionary method to code state legislation from 1991-2018 that would code a bill about a “learning environment” to be about environmental policy, and not education policy. Therefore, we instead take a “word embedding” approach, which is more nuanced than looking for the presence of words. The principle intuition behind word embedding is that “you shall know a word by the company it keeps”^3^ and has become the dominant approach to natural language processing (or NLP)^4^.

Specifically, we constructed a machine learning model based on the transformer architecture^5^, including the *Bidirectional Encoder Representations from Transformers* (BERT)^6^, and two other similar algorithms^7,8^. These models observe more than just keywords, utilizing contextual word-piece embeddings to build document embeddings to make sophisticated estimates of the proper category each bill could fit in. We then extend the classification system’s coverage by forming predictions about the policy content of bills for which no keywords are present. We also train the model to be capable of “doubting” the keywords themselves, allowing it to question homonyms like “learning environment,” which might otherwise map to the “Environment” topic, instead allowing it to be coded as “Education.” We iterate the model several times, overweighting model predictions with no keywords present so that the model does not just emulate the dictionary method.

Our method produces policy sector estimates for 1.36 million bills, which is the universe of bills in the Legiscan database, which itself has collected bills from state legislative websites since about 2009. We allow for an individual piece of legislation to be measured as pertaining to more than one subject. This is necessary because of cases where the name of the bill obscures its policy content, like Wisconsin’s 2011 “Budget Repair Bill” which gained national recognition for reducing the collective bargaining rights of state employees, or Minnesota’s 2016 “Omnibus Tax Bill” that also concerned hosting a World’s Fair and the Super Bowl^9^. To account for the fact that bill titles can be misleading on their own, we also use bill descriptions provided by Legiscan to feed the model.

We validate our estimates against published datasets to favorable results. This is not a straightforward task, as we are using a codebook that is backwards compatible with many studies on American state politics published by Virginia Gray, David Lowery and coauthors^10–13^ and other recent investigations of American legislatures^2,14^ but for which there is no “ground truth” data. Evaluating the model against a hand-coded sample of 1,000 bills, the model achieves a “Top-K agreement” of 80 percent, for K=3. This step is necessary for comparing a multi-label classification scheme to another multi-label classification scheme, but 80 percent is notable because that is the same target that human coders are trained to achieve on subtopics of the Comparative Agendas Project^15^. For an external validation, we investigate how the estimates compare to the hand-coded Pennsylvania Policy Database Project (PPDP)^16^. The machine learning estimates produce superior results compared to the legacy dictionary method reported by Garlick (2023)^2^ by matching the accuracy of the legacy method while offering far more coverage of the agenda. Knowing this reliability, there are advantages to our approach, especially its scalability. It is reproducible as well as cost- and time-effective, so researchers can take heart knowing that future projects can use this approach.

We offer suggestions for how best to use these estimates to observe and analyze state agendas. The estimates can be used to create a panel dataset that facilitates clear inter-state and inter-temporal comparisons. However, there is still considerable variation in how states behave, so it is best to group legislative sessions into two-year periods, the vast majority of which begin in odd-years following even-year elections. We detail the exceptions to this pattern, and also which bills and sessions to include in a typical analysis.

## Methods

Classifying state legislative text provides researchers with a number of tradeoffs. Researchers want data to be backwards compatible with extant studies, while also accounting for recent developments in society. Bills should be coded in a reliable way, while also acknowledging the reality that many bills do not concern a single subject area. This section summarizes our approach which accounts for these tradeoffs. We start by running a legacy keywords version of the model, closely approximating the approach used by prior researchers^2^. Then we introduced our machine learning model that (1) takes advantage of best in class algorithms to pre-process text in bill descriptions made available by Legiscan into “word embeddings,” (2) employs a novel “decision function” for determining if a bill fits each policy category.

Our model’s decision function *f*(*X*) internalizes the input data *X* and generates a prediction $\widehat{y}$. In short, the researcher who fits a classification system to legislative text to predict bills’ policy content applies the following workflow:

Read the input data *X* → Apply a classification decision function *f*( ⋅ ) → Generate $\widehat{y}$

### Finding and sorting data

The first step for creating the new dataset is to collect a corpus of state legislation. Beginning in the early 2000s, states began publishing their legislative records on the internet. However, the states vary a great deal in how detailed that information is, and how it is presented. A number of private and quasi-public efforts were launched to collect and centralize this data, most notably Legiscan and the OpenStates project. Legiscan is a private offering launched in 2010 and publishes bill titles, descriptions, and a 5-step bill history. OpenStates began as an open-source project before being purchased and maintained by the private firm Plural in 2021. They differ slightly in their offering, for example Legiscan has a 5 step bill progress variable, while OpenStates tracked detailed bill histories, which have been used to establish a 23-step bill histories. This study uses the bill descriptions and titles from Legsican for the universe of bills from 2009-2020, for a total of 1,360,994 bills. The full text is available, but legislation is often not helpful, as it is full of boilerplate language and references to legal code^17^.

We then set the destination policy categories, with a codebook with broad policy areas in state politics that occupy intuitive conceptual domains such as “Healthcare,” “Education,” and “Tax Policy”^10,18^. We did this by expanding upon the classification system with 23 topics used in the legacy model^2^, including 5 additional topics to improve its overlap with the Comparative Agendas Project spearheaded by Baumgartner and Jones, producing a multi-label system with ∣*K*∣ = 28 relatively broad policy areas.

We append several additional keywords to the topics listed in the legacy model^2^ to improve the classification system’s overlap with the Comparative Agendas Project codebook. The additional topics are “Fiscal and Economic Issues”, “Immigration”, “Public Lands and Water Management”, “Foreign Trade”, and “International Affairs and Foreign Aid,” which makes the scheme more complete and comparable to the Comparative Agendas Project. Table 1 presents this set of keywords and topics.

**Table 1:** Extended list of keywords for dictionary method.

Code	Codebook^*^		Prediction	Ground Truth
	GL	PAP		
G0100		*✓*	Fiscal & Economic	inflation, inflationary, unemployment, fiscal
G0201	*✓*		Civil Rights	civil right, civil rights, discrimination discriminated, discriminatory
G0205	*✓*		Environment	environment, environments, environmental environmentalist, environmentalism, environmentalists global warming, greenhouse gas, greenhouse gases, climate change
G0207	*✓*		Religion	church, churches, mosque, mosques, synagogue, synagogues religious, religion, religions
G0208	*✓*		Tax Policy	tax, taxes, taxed, taxable, taxing, taxation
G0300	*✓*		Health	health, healthcare
G0400	*✓*		Agriculture	agriculture, agricultural, farm, farmer, farmers, crops
G0500	*✓*		Labor & Employment	labor, laborer, laborers, laborers’, laborer’s, employee employees, employees’, employee’s, unions
G0600	*✓*		Education	education, educational, educations, educated, educate educates, educator, educators
G0701	*✓*		Utilities	utility, utilities
G0702	*✓*		Natural Resource	gas, gases, natural gas, oil, mineral, minerals natural resource, natural resources
G0900		*✓*	Immigration	immigration, immigrant, immigrants, undocumented, migrant migrants, migration
G1000	*✓*		Transportation	highway, transit, airport, highways, airports transportation
G1200	*✓*		Law	legal, legality, illegal, illegality, lawful, unlawful
G1300	*✓*		Welfare	social service, charity, charities, social services charitable
G1400	*✓*		Construction	construct, constructs, construction
G1500	*✓*		Bank	banking, real estate, financial institution interest rate, financial institutions, interest rates
G1502	*✓*		Small Business	retail, retailer, retailers, small business small businesses
G1503	*✓*		Sports	sport, sports, recreation, recreational
G1510	*✓*		Insurance	insurance, insure, insures, insured, insurer, insurers
G1520	*✓*		Manufacturing	manufacturing, manufacturer, manufacturers
G1600	*✓*		Military	military, veteran, veterans, veteran’s, veterans’ armed forces, army, coast guard, national guard, air force, navy marine corps
G1700	*✓*		Communication	media, telecommunication, telecommunications, telecom telecoms
G1800		*✓*	Foreign Trade	foreign trade, tariff, tariffs, export, exports
G1900		*✓*	Int’l Affairs and Foreign Aid	foreign aid, embassy, embassies, diplomat, diplomats
G2100		*✓*	Public Lands & Water Management	state park, state parks, state forest, state forests public land, public lands, water management
G2400	*✓*		Local Government	municipality, public employee, municipalities local government, county of, counties of, township, village(s) of towns of, city of, town of, cities of, fire protection district fire protection districts, fire district, fire districts
G2401	*✓*		Police and Fire	police, fire, peace officer, peace officers law enforcement, firefighter, firefighters, fireman, firemen, firewoman firewomen

^*^Originated in the Gray and Lowery codebook^2,10^, or the original Policy Agendas Project^16,18^.

We then replicate the legacy dictionary model used by Garlick (2023)^2^ that places bills in a policy category if they contain any of the listed keywords. This method is tried and true to reduce false negatives; however, even with the extended codebook, the legacy dictionary approach only places approximately 41% of the universe of state legislation into a policy category. A bill which mentions “medical” and “doctor,” for example, is not classified as “Health” unless it also had the precise keyword: “health.” Words which strongly *relate* to topics but are not keywords themselves have no effect on the output of this decision function. We will overcome these problems with a pre-processing approach that treats naturally occurring language more dynamically, and a three-pass decision function that emphasizes contextual clues, and not just keywords, to produce better coverage of the agenda without sacrificing accuracy.

### Transforming text to data

Machine learning models have moved beyond the unordered “bag-of-words” approach that was once common in political science^19^, but served as sufficient for dictionary models^2^. But meaning is lost when word order is not taken into account. A bill that reads, “this bill is about taxes - not education,” has an identical bag of words to, “this bill is about education - not taxes”, and bags of words approaches are also vulnerable to homonyms, as our prior example “the learning environment” might fall under the purview of an “Education” topic, but “learning about the environment” might be better fit under “Environment” (or, “Education” and “Environment” jointly).

Word embedding presents a solution, as words are represented as real-valued, *N*-dimensional vectors whose elements reflect latent semantic dimensions that, together, define the position the word occupies in an *N*-dimensional space - its “meaning.” For example, one might expect the words “teacher” and “student” to be more semantically similar than “teacher” and “warfare.” In this way, the word embedding approach can drastically reduce the dimensionality of the input data. Instead of representing every word as a unique token ID, every word, and the document itself, is represented in a common *N*-dimensional space shared by all documents, sentences within documents, and words within sentences. Therefore, the elemental unit of analysis in a word embedding model is the “word-piece”^20^, where e.g. the word-piece “educat” and “##ion” have their own word-piece embeddings, with the “##” tag indicating that “ion” should be attached to the word-piece that comes before it. Popular algorithms that generated static estimates for embeddings include Word2Vec^21^ or GloVe^22^.

A recent innovation from the static models has been greater focus on the context surrounding word embeddings. The transformer model^5^ utilizes a “self-attention” mechanism, which allows the model to weight the importance of words in the input sequence as they pertain to the focus word. For example, consider the sentence: “she went to the *hardware store* to buy a *hammer*.” If a human were asked why “hammer” is a “logical” word to end the sentence - and why not “car,” “house,” or “bagel” - they would likely point to “hardware store” as the leading contextual clue. The word “hardware” attends to “store,” dynamically modifying its word embedding. If the word “hardware” were removed from the input sequence, the prediction of “hammer” might be less likely (unless the model were trained on relevant data), and the prediction of “bagel” might be more plausible.

The model we train to classify legislation into policy areas utilizes the transformer model BERT, *RobustBERT* or “*RoBERTa*”^7^, and XLNet^8^. These models utilize contextual word-piece embeddings to build document embeddings, which are the “input data” *X* that our model is trained on.

### Decision function

The first pass of our decision function is similar to using a dictionary method to assign bills to topics. It is a supervised multi-label classification task, and for each bill we look to classify, the model will match word embeddings with the destination topics in the training data. For example, like a dictionary model, if the term for the embeddings equivalent of “transit” is present, it would predict the topic “Transportation”  ∈ *y*. Dictionary methods are transparent but their deterministic nature likely generates too many false negatives, as well as posing a risk for false positives as well.

Next we need to acknowledge the limitations of dictionary methods, and generate predictions for bills for which no keyword-equivalent embeddings are present. In pursuit of this end, the model is our direct antagonist: if we simply train the model on the dictionary method-coded bills, the model could perfectly reverse-engineer the dictionary method, imposing the same issues of false positives and false negatives that dictionary models face. To train the model to learn a more nuanced representation of topics, we must block the deterministic dictionary path such that learning about the context surrounding keywords is the only way for it to make progress on its training task.

The second pass breaks the model’s ability to memorize keywords, by artificially corrupting the input data in several ways: (1) randomly replace words in bill summaries with synonyms; (2) randomly delete words from bill summaries; (3) randomly “mask” words in bill summaries (i.e. the model knows a word is there, but does not know what it is); and (4) mask *every* occurrence of keywords in bill summaries, e.g. the phrase “about the learning environment” would be corrupted to be read “about the learning [mask].” It is crucial to our procedure that we do not solely mask keywords, but randomly mask other words as well; otherwise, our model could infer the missing keywords due to the context that surrounds them, thereby reverse-engineering the dictionary method. So the absence of keywords forces the model to learn how the context surrounding keywords can help in predicting the bills’ topics.

The second pass of the model generates predictions for the bills for which no keyword-equivalent embeddings are present. From the standpoint of the original dictionary method, any bills which lack keywords but have topic predictions are technically “false positives,” but with a by-hand inspection, these model disagreements typically yield an accurate measure of the bill’s topic(s), therefore we treat these predictions as valuable, as they feature the bills with word embeddings apart from the dictionary-aligned approach. We will take advantage of these bills in our final pass.

In the third pass, the model is then trained from scratch on the bills from the second pass without keyword-equivalent embeddings. With the second pass predictions as the ground truth labels, we generate predictions for the bills for which keyword-equivalent embeddings *are* present. In summary, the model learns how to form predictions for bills which lack keywords by learning how the context surrounding keywords can be used in their absence (second pass). The model forms said predictions, and then is re-trained on its own predictions for bills which lack keywords from scratch (third pass). This severs the link between keywords and topics because, during the third pass, it is never exposed to the keywords.

The model’s produces a set of probabilities it associates with observing each topic. Researchers should set a cutoff value *τ*, above which to predict the presence, and below, the absence, of a given topic. In the results section to follow, we set *τ* = 0.5, but researchers could utilize other values for *τ* if they are more concerned the model’s *Precision* to produce fewer false positives and wherein a higher *τ* works better, or *Recall* to reduce false negatives with a lower *τ*).

## Data Records

The estimates are publicly available at two levels of specificity. First, the “individual bill” estimates^23^ contain the model’s confidence that the bill applies to each of 28 policy areas (or *τ*), which is 38.1 million estimates. The variables in this .csv file are an identifier that corresponds to the master set (**dg_id**), the model’s confidence that the bill suits each policy area (**tau**), and the policy codes in string format (**policycode**), which are described in Table 1.

As an example of the individual bill estimates, Table 2 provides the model’s output for West Virginia’s 2011 bill “Creating the ‘Health Care Choice Act.’” As the title would suggest, this bill has an extremely high policy prediction for Health (0.989 out of 1.0), however, it receives an even higher prediction for Insurance (0.999). The bill description indicates how this bill regulates insurers, which the model has used to determine it to be an insurance bill. No other policy areas remotely approach our default confidence level: *τ* = 0.50, so the model determines that this bill only applies to two policy areas.

**Table 2:** Model output for WV’s HB 2801 (2011) titled: “Creating the ‘Health Care Choice Act’”.

No.	Prediction	Label (Code)	Present	No.	Prediction	Label (Code)	Present
1	0.999	Insurance (G1510)	*✓*	15	0.015	Environment (G0205)	
2	0.989	Health (G0300)	*✓*	16	0.015	Small Biz (G1502)	
3	0.060	Trade (G1800)		17	0.014	Commun. (G1700)	
4	0.034	Int’l Affairs (G1900)		18	0.014	Police/Fire (G2401)	
5	0.029	Pub. Lands (G2100)		19	0.014	Labor (G0500)	
6	0.022	Immigration (G0900)		20	0.013	Construction (G1400)	
7	0.020	Tax (G0208)		21	0.012	Agriculture (G0400)	
8	0.020	Energy (G0702)		22	0.012	Sports (G1503)	
9	0.019	Local Gov’t (G2400)		23	0.011	Civil Rights (G0201)	
10	0.018	Manufacturing (G1520)		24	0.010	Welfare (G1300)	
11	0.018	Bank (G1500)		25	0.009	Macroecon (G0100)	
12	0.016	Religion (G0207)		26	0.008	Law (G1200)	
13	0.016	Transport. (G1000)		27	0.008	Education (G0600)	
14	0.016	Utilities (G0701)		28	0.006	Military (G1600)	

*Bill description*: A BILL to amend the Code of West Virginia, 1931, as amended, by adding thereto a new section, designated §33-44-14, relating to creating the “Health Care Choice Act”; and enabling insurers authorized to sell insurance coverage in selected states to engage in the business of insurance in West Virginia.

Second, there is a “master” dataset^24^ which reflects the policy areas that apply to each of the 1.36 million bills from approximately 2009-2020. Researchers can collapse this file to produce an estimate of state policy agendas. The variables in this .csv file include indicators (0,1) for each of the policy areas named in Table 1, as well as an identifier that corresponds to the individual estimates dataset (**dg_id**), the bill’s code (**ajo_id**), metadata on the bill drawn from Legiscan including its number (**legiscan_bill_number**), type (**legiscan_bill_type_verbose**), title (**legiscan_title**), how far the bill advanced on a five-point scale (**legiscan_status**), the session the bill was introduced in (**legiscan_session**), and the first (**legiscan_session_year_start**) and last (**legiscan_session_year_end**) years of the session. See the Usage Notes section for more details on these variables.

The confidence level in the master dataset is set to *τ* = 0.50, and 51 percent of bills have a single policy code, while 26 percent have two policy codes. Only 95 bills have more than five codes. The bills that have unusually high numbers of policy codes, like Montana’s 2021 HB2 titled “General Appropriations Act,” are usually omnibus legislation or appropriations bills regarding many state agencies.

**Fig. 1:**
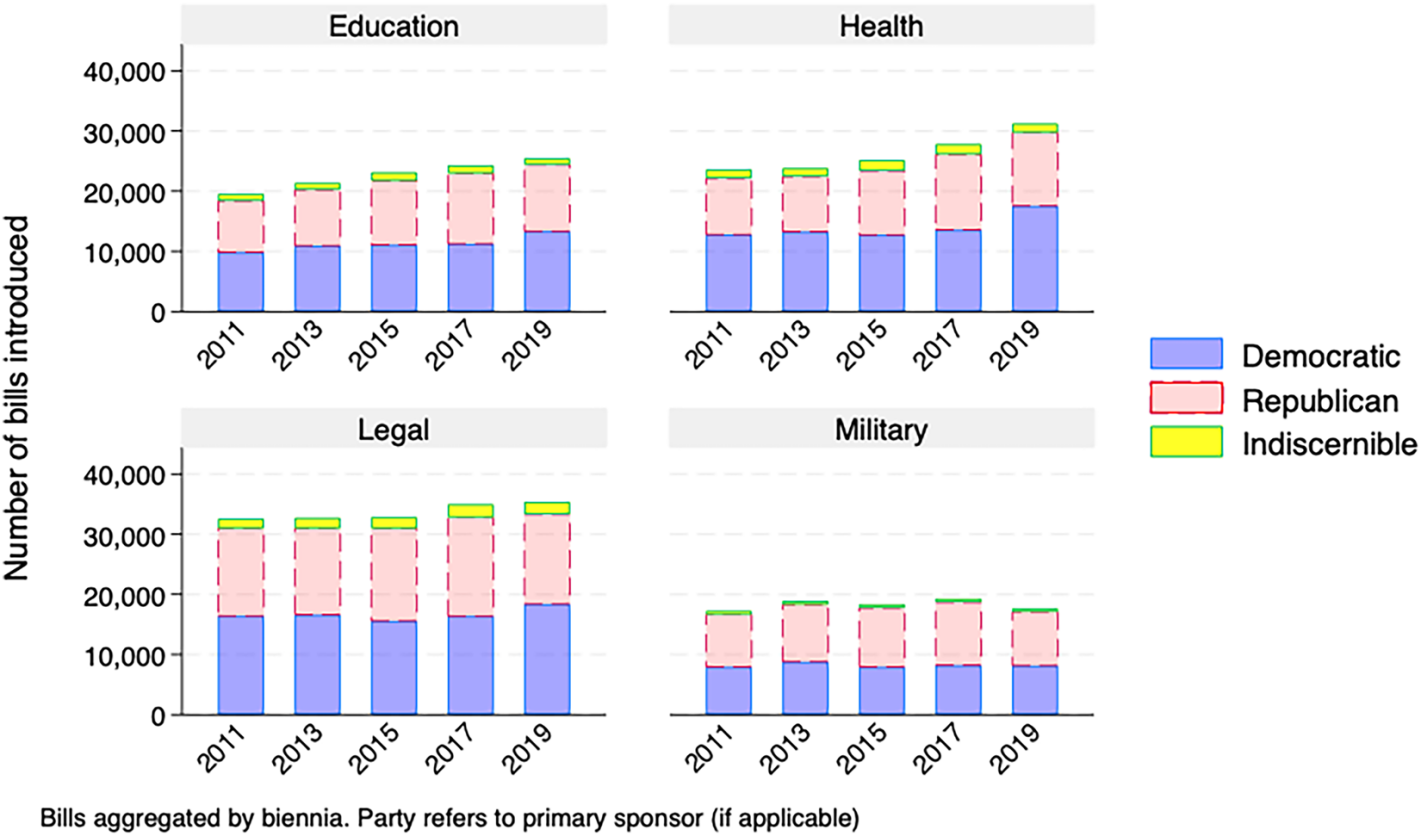
Number of bills introduced by biennia in the most populated policy categories (education, health, legal and military).

**Fig. 2:**
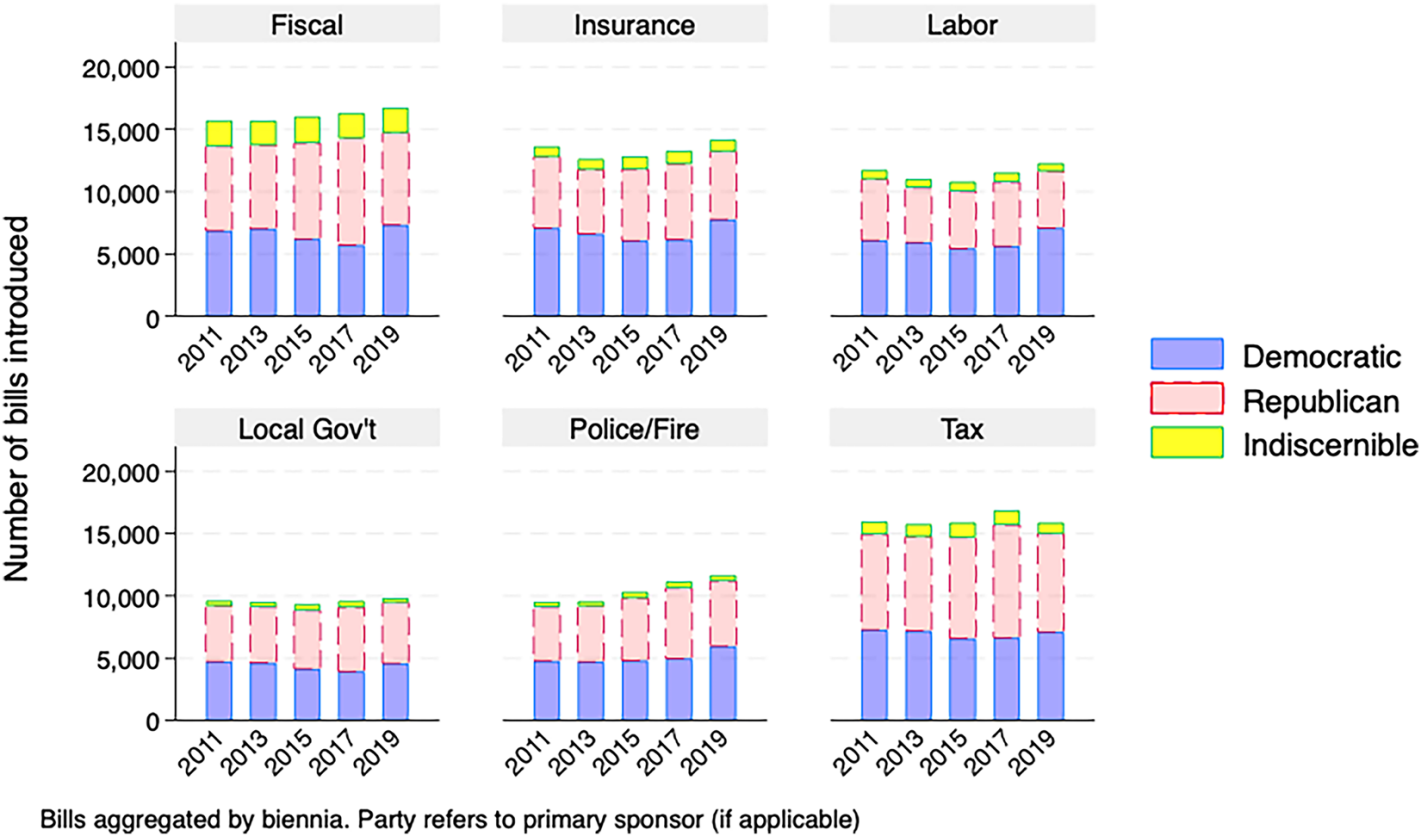
Number of bills introduced by biennia in highly populated policy categories.

**Fig. 3:**
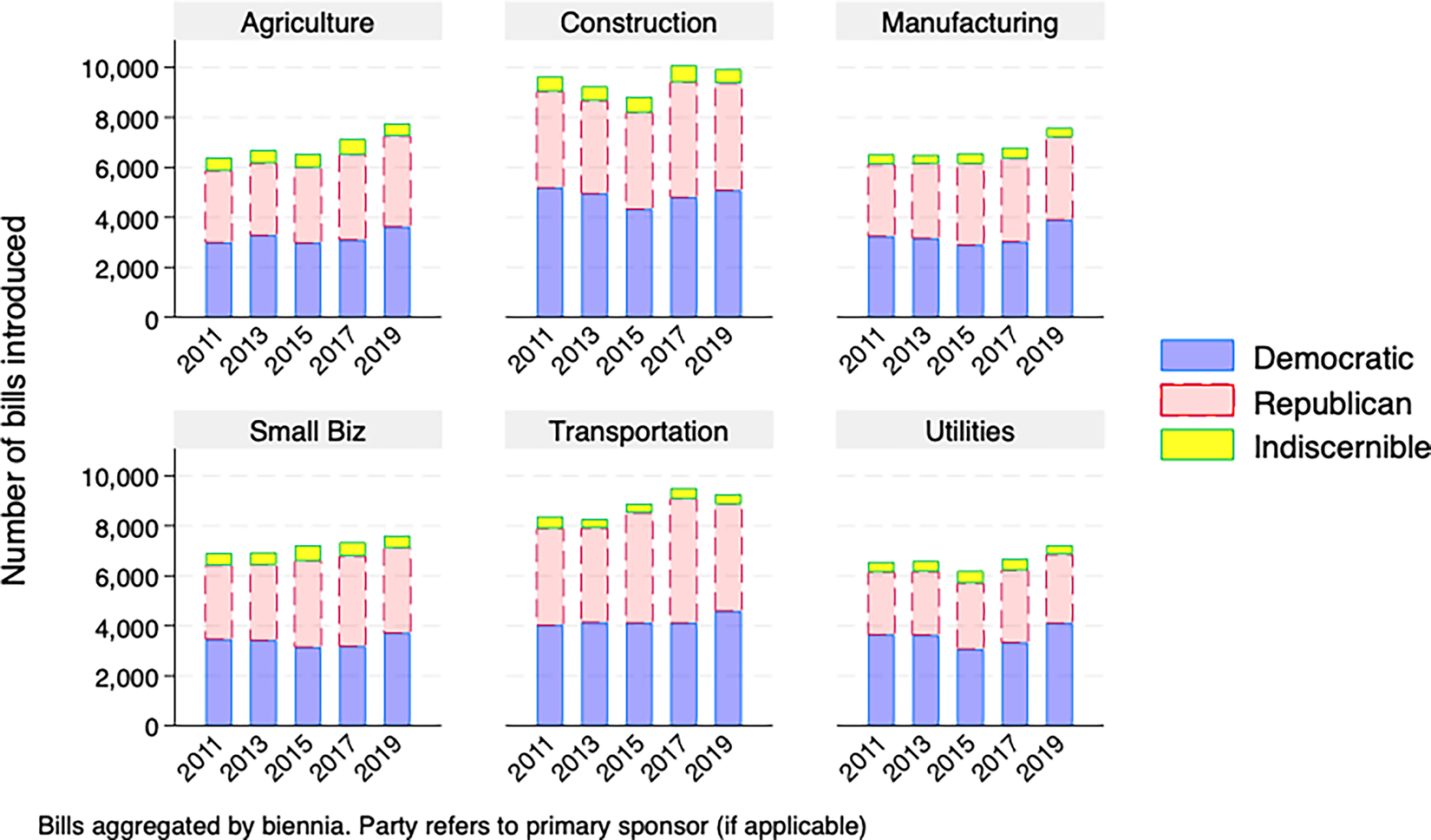
Number of bills introduced by biennia by policy categories.

**Fig. 4:**
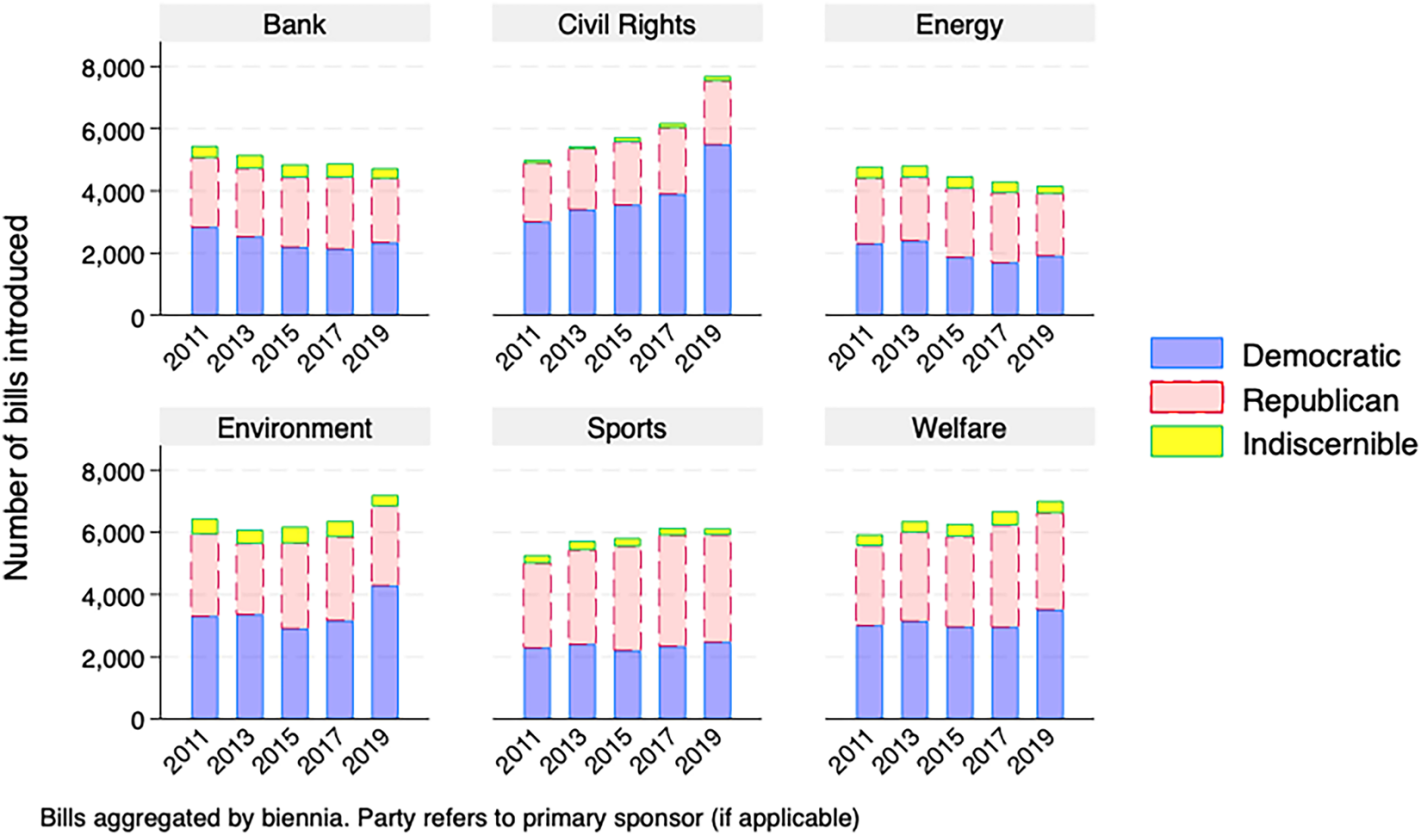
Number of bills introduced by biennia by policy categories.

**Fig. 5:**
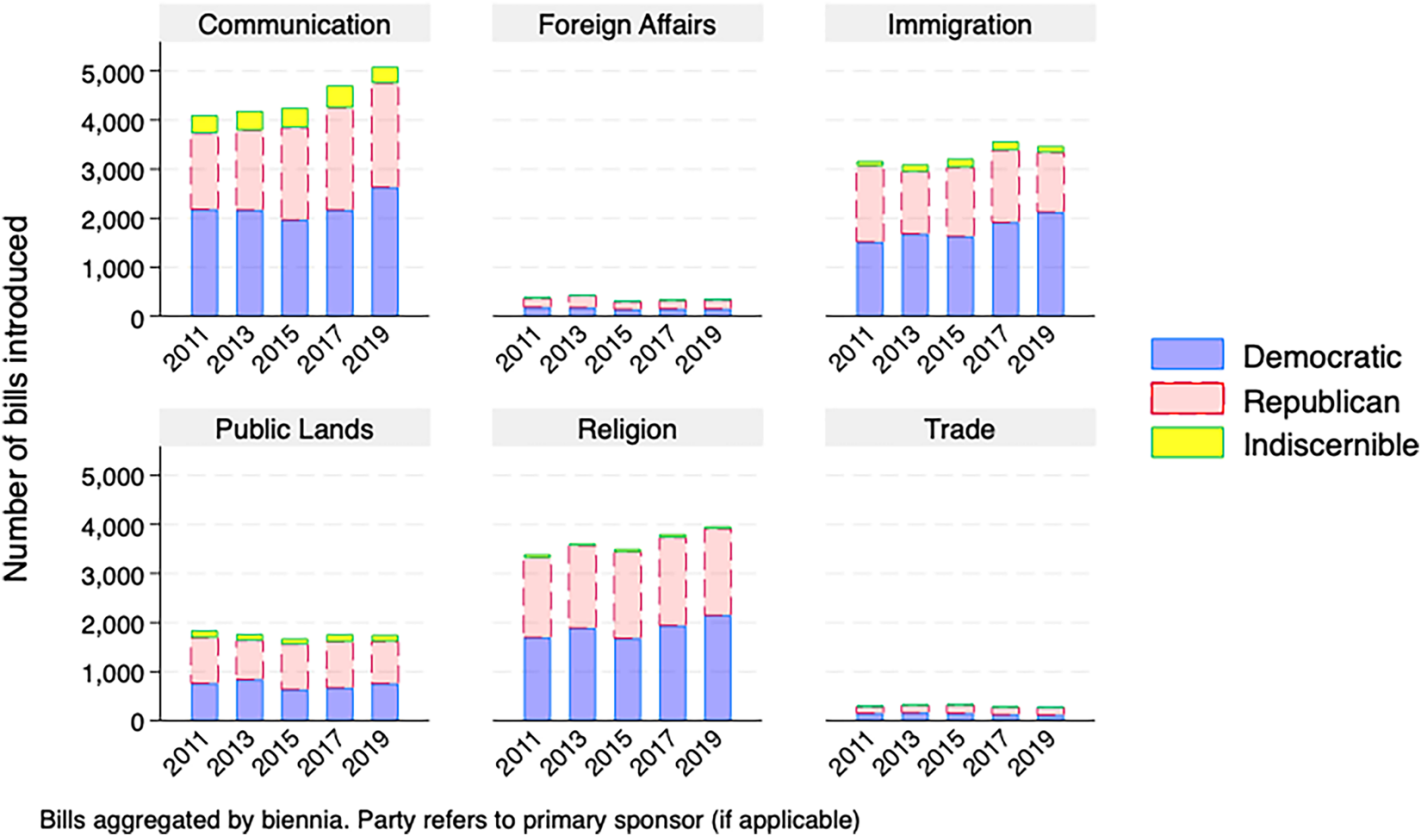
Number of bills introduced by biennia in the least populated policy categories.

For a descriptive overview of the master dataset, Figs. 1–5 demonstrate the number of bills assigned to each of the 28 categories from 2009-2019, aggregated by biennium and separated by the primary author of the bill, if it is discernible. While the amount of attention paid to many policy areas has increased over time, like how Fig. 1 shows for education and health, this has not been the case across the agenda, as Fig. 3 shows that less attention to sectors like banking over time. This follows as there was a societal fascination with banking at the beginning of the decade in the wake of the 2008 financial crisis that has faded. In addition to the changes over time, the levels are also important here. Our method has shown that the military, including veterans issues, is a major focus of state legislation that previous efforts have missed.

There are also notable partisan patterns, however it is worth noting that the raw count of bills being introduced is affected by how many Republican, Democratic or third-party/independent legislators are in office for a given session. With that caveat in mind, we see certain issues having most bills primarily authored by Democrats, like Civil Rights in Fig. 4 or Republicans, like Religion in Fig. 5.

## Technical Validation

There are challenges to validating these data, as there is no “ground truth” dataset of state legislative bills coded under the Gray and Lowery codebook. This presents two issues: first, translating between disparate codebooks introduces measurement error, as bills could be “correctly” assigned according to each codebook but not translate to each other. For example, the Gray and Lowery codebook has a category for electric utilities (“Utilities”) that is separate from the PPDP’s “Energy” topic, so any bill assigned to utilities could be labeled as incorrect. Second, the complexity of assigning bills to a single policy area is a demanding task on its own. Many state politics researchers have deemed the classification task complex enough to necessitate that a domain expert inspect documents by-hand. For example, Reingold *et al*. (2021)^25^ first applies a dictionary method to identify legislation concerning abortion by searching for the “abortion” keyword, and then their second stage of analysis requires hand-coding bills to measure their abortion “sentiment.” The Congressional Bills Project^1^ and the codebook to which it adheres do away with keywords entirely, instead training hand-coders for several weeks to identify the leading policy area of the bill. This approach has its own issues, first it is not 100 percent reliable. Hillard *et al*. (2008, p. 40)^15^ report that coders are trained until they code congressional bills by 21 major topic codes at 90 percent reliability, and the 200+ minor topic codes at 80 percent reliability. Second, using hand-coders comes at the expense of transparency; unless the project clearly annotates the “why” for each hand-coder classification decision, it is difficult to diagnose hand-coder versus downstream researcher disagreements.

To address these concerns we take the following steps to demonstrate the validity of these estimates. First, we compare the machine learning model’s estimates to the legacy dictionary method. This shows how the machine learning model offers similar levels of precision, which provides confidence that the model is finding the policy content it is claiming to, but far better recall, or the degree the model is finding all possible bills in each policy area. Second, we compare the model to estimates generated by hand-coders^16^ to show that the model is reliable.

These exercises demonstrate the external validity of these estimates such that machine learning estimates track the hand-coded estimates of the Pennsylvania legislature closely over time. In particular, the machine learning greatly outperforms the legacy dictionary method in its recall of potential bill codings.

### Model Evaluation Against Legacy Dictionary Method

We first evaluate the machine learning estimates against the legacy dictionary method. This is not a traditional validation, as the dictionary method is less of a “ground truth” than an alternative approach. But by showing which policy areas these two models agree and disagree on, we can better understand how the machine learning model operates. In Table 3, a “false positive” denotes an instance where the model predicts the presence of a given topic despite the absence of all its keywords. A “false negative” denotes an instance where the model predicts the absence of a given topic despite the presence of one of its keywords. “True positives” and “true negatives” are cases where the model and dictionary method agree to assign or not assign the topic to the bill, respectively.

**Table 3:** Model performance evaluated against the legacy dictionary method.

Topic	*True*	*False*	*True*	*False*	Precision	Recall	*F*_1_ Score
	*Pos*.	*Pos*.	*Neg*.	*Neg*.			
Tax Policy	75,058	26,288	1,222,541	37,107	74	67	70
Foreign Trade	4,188	3,573	1,352,506	727	54	85	66
Transportation	28,825	25,922	1,293,531	12,716	53	69	60
Police and Fire	32,875	31,386	1,285,187	11,546	51	74	60
Labor and Employment	36,349	37,616	1,265,878	21,151	49	63	55
Local Government	26,830	29,715	1,281,215	23,234	47	54	50
Health	70,380	96,796	1,178,041	15,777	42	82	56
Agriculture	17,401	25,952	1,313,319	4,322	40	80	53
Insurance	33,912	49,878	1,269,258	7,946	40	81	54
Education	52,843	87,123	1,194,764	26,264	38	67	48
Natural Resource	9,616	19,233	1,322,858	9,287	33	51	40
Utilities	12,632	28,405	1,318,188	1,769	31	88	46
Military	36,180	81,761	1,238,984	4,069	31	90	46
Environment	13,009	30,954	1,310,959	6,072	30	68	41
Religion	6,229	16,709	1,337,153	903	27	87	41
Construction	15,286	42,622	1,298,526	4,560	26	77	39
Public Lands and Water Management	3,240	9,183	1,347,280	1,291	26	72	38
Bank	7,392	25,410	1,325,243	2,949	23	71	34
Small Business	10,169	34,779	1,311,516	4,530	23	69	34
Fiscal and Economic Issues	20,419	79,291	1,253,791	7,493	20	73	32
Sports	5,986	29,289	1,322,176	3,543	17	63	27
Immigration	3,935	19,594	1,337,063	402	17	91	28
Civil Rights	5,089	33,152	1,321,595	1,158	13	81	23
Welfare	4,900	34,107	1,320,355	1,632	13	75	22
Manufacturing	6,006	38,707	1,314,548	1,733	13	78	23
Communication	3,625	24,991	1,330,391	1,987	13	65	21
Law	11,438	191,236	1,149,966	8,354	6	58	10
International Affairs and Foreign Aid	216	4,322	1,356,431	25	5	90	9
Macro-average					31	74	40
Micro-average					34	72	43

Notes: Model performance is evaluated against the dictionary method, i.e. a “true positive” (*TP*) denotes an instance where there is a keyword present *and* the model concurs with the topic prediction. A “false positive” (*FP*) denotes an instance where there is not a keyword present, but the model predicts the topic regardless. A “true negative” (*TN*) denotes an instance where neither the keyword is present, nor does the model predict the topic. A “false negative” (*FN*) denotes an instance where the keyword is present, but the model does not predict the topic. Macro average is computed with each policy area equally weighted, and micro-average is computed with policy areas weighted by their respective number of bills coded.

For almost all topics, false positives outnumber true positives, owing to the fact that the legacy dictionary method generates codes for a much smaller share of bills. A higher topic *Precision* denotes a larger share of true positives compared against all positives; as *Precision* increases, the model finds fewer instances where it predicts the presence of a topic despite the absence of keywords, meaning the keywords are more “necessary” for identifying the topic. For example, the most precisely-defined topic, by far, is “Tax Policy”, with *Precision* = 74%. It may be hard-pressed to think of a bill pertaining to “Tax Policy” which lacks all mentions of “tax,” “taxation,” “taxable,” and so-on (or at least, it may be harder to find such instances than it would be to find counterparts in other topics), but there are “Tax Policy” bills that include mentions of a “levy,” “Department of Revenue,” “economic development,” or “bonds.”

The lowest precision topics are “International Affairs and Foreign Aid” (5%), “Law” (6%), and “Civil Rights” (13%), meaning the model frequently predicts the topics’ presence without keywords being present. To shed light on whether these overrides of the dictionary method’s keyword rules are valuable, “International Affairs and Foreign Aid” (abbreviated in the table as “Intl. Affairs”) often include “exchange programs” (for students), explicitly mention other countries, or include other nouns plausibly associated with international affairs. For example, the dictionary defined in Table 1 includes the keywords “diplomat” and “embassy,” but not “ambassador,” and yet, “ambassador” takes on a similar semantic meaning with respect to the machine learning model’s classification task. Using the keywords associated with the “Civil Rights” topic as its conceptual “seeds,” the model has grown the topic to include a large number of resolutions, especially those which “celebrate the life” of an individual, mention “slave trade,” “equal opportunity,” or “human rights.” The multi-label nature of the data proves especially useful in flagging e.g. a bill which mentions “Female Veteran’s Day” as pertaining to both “Civil Rights” and “Military,” and a bill mentioning the “Equal Opportunity Scholarship Act” as pertaining to both “Civil Rights” and “Education.” False positives for “Law” often discuss “liability,” “unenforceable,” “covenant,” “contract,” “Juvenile Justice,” and “allegations.”

**Fig. 6:**
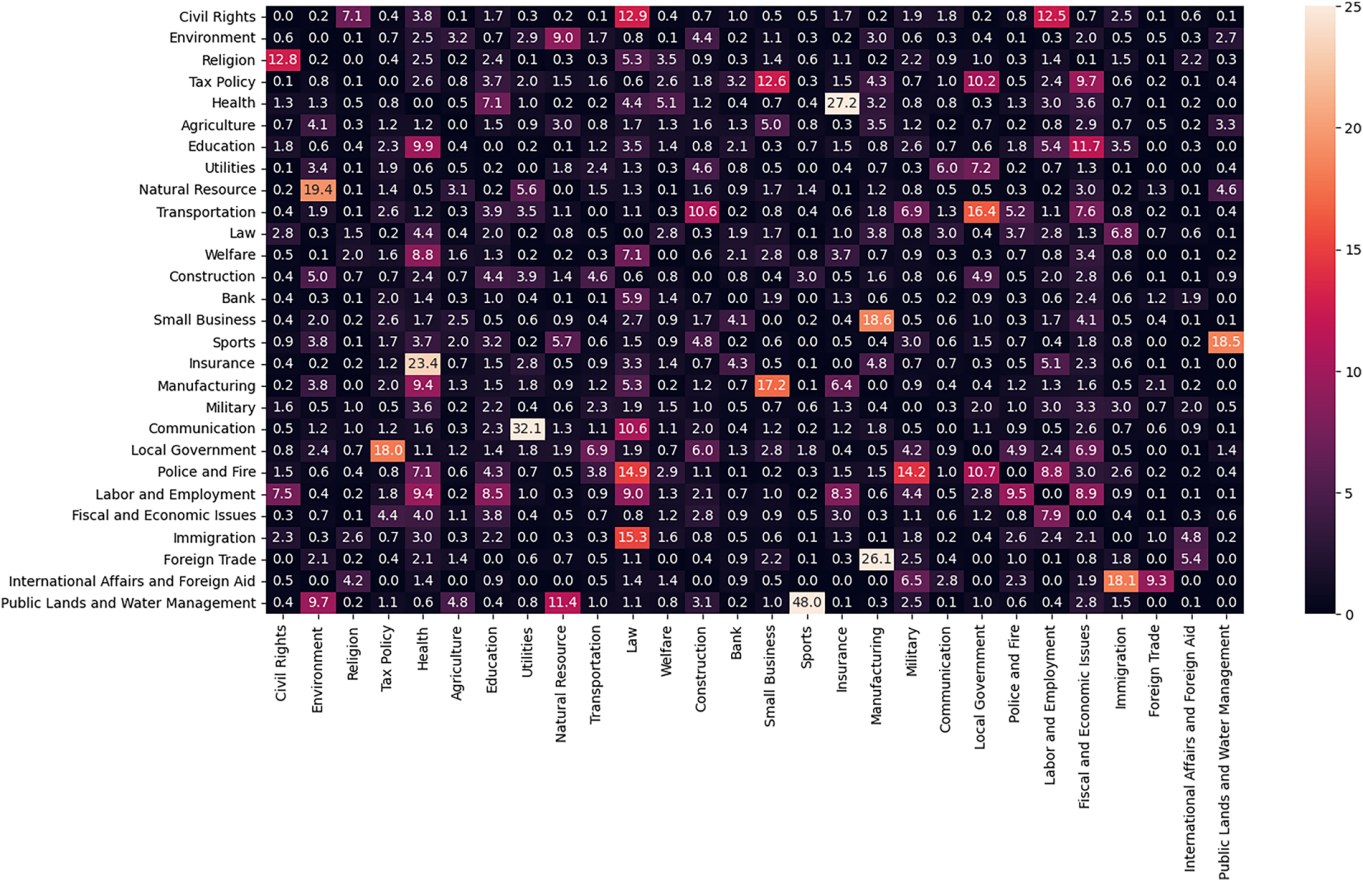
Correlation between topics based on model predictions, no keywords. Notes: Cells denote the frequency with which bills predicted as pertaining to *Topic* = *Row* are also coded as *Topic* = *Column* by the model, observing only bills for which there are *no* keywords present from either *Topic* = *Row* or *Topic* = *Column*. For example, 12.8% of bills which were predicted as pertaining to “Religion” (while lacking any “Religion” keywords) were also coded as pertaining to “Civil Rights” (while lacking any “Civil Rights” keywords).

To provide a high-level perspective the internal consistency of the model, Fig. 6 presents a heatmap of topic co-occurrences for bills for which no keywords are present. Using non-keyword-coded bills illustrates that how the model performs without the strongest clues regarding policy areas. For each of the figures, a cell value denotes the percentage of bills coded as *Topic* = *Row* which were jointly coded as *Topic* = *Column*. For example, the “Civil Rights” topic most frequently co-occurs with “Religion,” “Law,” and “Labor and Employment,” even when of whether the bills contain zero keywords. The other top co-occurrences are “Environment” and “Public Lands and Water Management” and “Sports” (often with references to fishing and hunting), “Health” and “Insurance,” “Foreign Trade” and “Manufacturing,” “Construction” and “Utilities,” as well as “Natural Resource” and"Environment.” The “Utilities” topic most often co-occurs with “Communication” (e.g. telecomms) and “Local Government.”

### Internal Validation

To demonstrate the face validity of the data, we drill down into the estimates with an issue that has emerged in the years since the original dictionary codebooks were published: fracking. Fracking developed as a political issue in the US following the development of “hydraulic fracturing” or “hydrofracking” technology in the mid-2000s allowed for energy companies to take advantage of shale underneath many, but not all, American states to produce natural gas and oil. While this was a revolutionary technique for energy extraction, it also had concerning environmental consequences, as the chemicals used for fracking, and its byproducts, could be toxic for groundwater and other environmental factors.

**Fig. 7:**
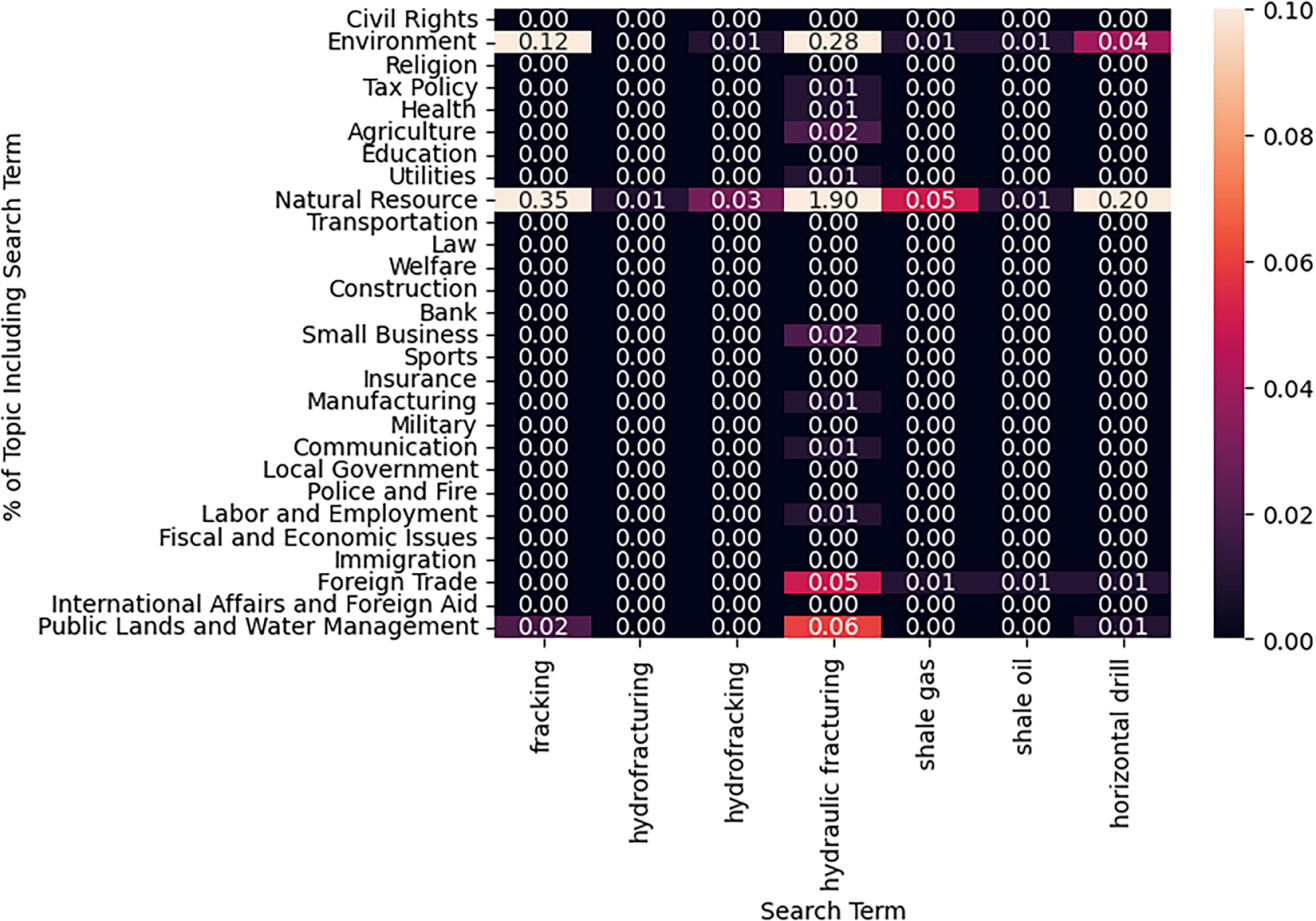
Topic hit rate for example search terms related to “Fracking.” Notes: A cell denotes the percentage of bills predicted to be assigned to *topic* = *row* that include the (case-insensitive) search term associated with its column.

To test for fracking being accounted for by our model, we look to see if bills containing the many synonyms for this new technology are coded in the categories we would expect. We identify fracking bills using the following (case-insensitive) search terms in bill titles and descriptions: *fracking, hydro fracturing, hydro-fracturing, hydrofracturing, hydro fracking, hydro-fracking, hydrofracking, hydraulic fracturing, hydraulic-fracturing, shale gas, shale oil, horizontal drill, horizontal gas, horizontal stimulation, horizontal well, fracturing fluid, fracturing wastewater, fracturing water, and fracturing chemical*. At least one of these search terms appeared in 736 bills from 2009-2020. We find that the model assigns them in logical categories. Figure 7 illustrates that the fracking-related search terms are mostly concentrated to the “Energy or Natural Resource” and “Environment” topics, there is also some prevalence in “Public Lands and Water Management”. We take this exercise as further evidence’s of the model’s internal reliability.

A human coder also evaluated a sample of 1,000 randomly selected bills drawn from a separate database (OpenStates) to 1) evaluate the completeness of the sample drawn from Legiscan, 2) allow for the comparison of a multi-class system, 3) provide feedback on the uncertainty of the process. This exercise produced a number of results. First, it showed that Legiscan contained 100 percent of the bills in the OpenStates archives, which reaffirms our decision to take the Legiscan data at face value. Second, the human coder revealed the difficulty of these coding decisions. The human coder was provided the same bill title from legiscan that the model was fed, and was asked if this was sufficient to assign a bill or if it was necessary to look up the full text of the bill via a link to its pdf. On 25.4% of bills, they required the full text. This was not data that the model had access to. Third, they were instructed to place the bill into a single category, if feasible, or if the bill was too complex, to assign a second category. They found that 44% of bills required a second category.

We evaluate the accuracy of the model in two ways designed to evaluate multi-label prediction models^26^. First, we estimate the model’s “Ranking Loss”, which calculates the average number of incorrectly labeled pairs. For example, the hand-coder assigned Alabama’s “Property Insurance and Energy Reduction Act” (SB 220 from 2015, titled: “Energy efficiency projects, financing by local governments authorized, non ad valorem tax assessments, liens, bonds authorized, Property Insurance and Energy Reduction Act of Alabama”) as “Natural Resources” and “Local Government”, but the model’s first two estimates were “Utilities” (*τ* = 0.98) and “Tax Policies” (*τ* = 0.42). Since “Local Government” (*τ* = 0.16) was the third highest estimate, the ranking loss for this bill was 2. A perfect classifier would be zero, and chance would be 0.5. The average ranking loss for this sample of bills was 0.043, an impressive score.

Another way to conceive of multi-label classifier is through Top-K agreement, which indicates the share of times the hand-coder assigns the bill to a variable (K) number of classes. The previous example would be a bill that does not find agreement at K=1 or K=2, but does find agreement at K=3. Table 4 shows how if only the top option is included, there is agreement between the human coder and model estimates on 57 percent of bills. If the first three model estimates are considered there is agreement on 80 percent of bills. This is similar to the Policy Agenda Project’s considers to be suitable for human coder performance on minor topic codes.

**Table 4:** Top-K agreement of model estimates and human coded sample (n=1,000).

K	Agreement	K	Agreement
1	0.57	6	0.88
2	0.74	7	0.89
3	0.80	8	0.91
4	0.84	9	0.92
5	0.86	10	0.93

The accuracy and precision of the model can also be calibrated using different *tau* cutoff rates. Table 5 shows the range, and the default level (*τ* = 0.5). A higher tau would only provide more confident estimates, at the expense of total coverage. The lower F1 scores can be considered an artifact of the multi-label to multi-label comparison.

**Table 5:** Evaluation of machine learning estimates (at different levels of *τ* against human coded sample (n=1,000).

*τ*	Precision	Recall	F1
0.9	0.59	0.23	0.31
0.8	0.55	0.25	0.32
0.7	0.52	0.30	0.36
0.6	0.49	0.32	0.37
0.5	0.45	0.41	0.39^*^
0.4	0.42	0.49	0.41
0.3	0.41	0.54	0.44
0.2	0.37	0.61	0.43
0.1	0.27	0.69	0.38

^*^Default level of *τ* used to generate estimates.

### External Validation

The PPDP provides an opportunity for an external validation of our estimates. The PPDP was handcoded by researchers using a version of the Comparative Policy Agendas codebook, adjusted for the context of state politics. The PPDP coded the universe of Pennsylvania legislative bills from 1979-2016, and the manual nature of this process has been considered the gold standard in the field before the implementation of automated methods. However, there are two barriers to comparing these two sets of estimates of the same bills before the Pennsylvania legislature. First, as shown in Table 6, there is misalignment on some topics. The Comparative Policy Agendas codebook was designed to account for the issues dealt with by national leaders of western democracies, including topics like “Macroeconomics” or “International Affairs and Foreign Aid” that are not relevant for a codebook optimized to study American state politics^10,27^. Therefore, we use 17 policy areas that overlap across the two datasets: civil rights, health, agriculture, labor, education, environment, energy, transportation, legal, welfare, construction, military, communications, public lands, local government. There are some difficult decisions to be made aligning these coding schemes, and we err on the side of caution by not assuming that the PPDP’s “Domestic Commerce” code would include several different codes that could fit there including: “insurance”, “manufacturing”, “bank”, or “small business.”

**Table 6:** Alignment between PPDP and machine learning codebook.

No.	Major Topic	GL Topic	Code
1	Macroeconomics		
2	Civil Rights and Liberties	Civil Rights	G0201
3	Health	Health	G0300
4	Agriculture	Agriculture	G0400
5	Labor	Labor and Employment	G0500
6	Education	Education	G0600
7	Environment	Environment	G0205
8	Energy	Natural Resource	G0702
9	Immigration	Immigration	G0900
10	Transportation	Transportation	G1000
12	Law and Crime	Law	G1200
13	Social Welfare	Welfare	G1300
14	Housing	Construction	G1400
15	Domestic Commerce		
16	Defense	Military	G1600
17	Technology	Communication	G1700
18	Foreign Trade	Foreign Trade	G1800^*^
19	International Affairs	International Affairs and Foreign Aid	G1900^*^
20	Government Operations		
21	Public Lands	Public Lands and Water Management	G2100
24	Local Government and Governance	Local Government	G2400
		**Unmatched codes**	
		Fiscal and Economic Issues	G0100
		Religion	G0207
		Tax Policy	G0208
		Utilities	G0701
		Bank	G1500
		Small Business	G1502
		Sports	G1503
		Insurance	G1510
		Manufacturing	G1520
		Police and Fire	G2401

^*^Conceptually aligned, but this was excluded because there were zero matches with the legacy model.

The second barrier to comparison is that the PPDP puts bills into only one major topic area and our model estimates a number of policy labels for each bill. This puts a ceiling on the potential precision of our measure. For example, our model estimates that 2009’s House Bill 890 “Establishing a nursing and nursing educator loan forgiveness and scholarship program” is coded as “Health” and “Education,” while the PPDP only considers it to be an “education” bill. Therefore, it is a “false positive,” while we consider this bill aimed at reducing the nursing shortage to bill a health care bill. Interestingly, if we only use a dictionary model based on the presence of keywords, that bill is also only labeled as “Education”. As above, we will compare both the keyword-only estimates as well as the modeled estimates in Table 7.

**Table 7:** External validation of modeled estimates using the Penn. Policy Database Project: 2007-2016.

No.	Major Topic	Machine Learning Estimates			Legacy Keyword Model		
		Precision	Recall	F1	Precision	Recall	F1
2	Civil Rights	0.42	0.27	0.33	0.58	0.10	0.17
3	Health	0.67	0.77	0.72	0.73	0.45	0.55
4	Agriculture	0.44	0.52	0.47	0.59	0.41	0.48
5	Labor	0.24	0.50	0.32	0.18	0.23	0.20
6	Education	0.60	0.70	0.65	0.53	0.34	0.42
7	Environment	0.61	0.38	0.47	0.53	0.15	0.24
8	Energy	0.47	0.41	0.43	0.58	0.39	0.47
10	Transportation	0.43	0.37	0.40	0.41	0.20	0.27
12	Legal	0.56	0.62	0.59	0.22	0.02	0.04
13	Welfare	0.21	0.33	0.26	0.70	0.10	0.18
14	Construction	0.15	0.26	0.19	0.29	0.18	0.22
16	Military	0.42	0.88	0.57	0.75	0.71	0.73
17	Communications	0.10	0.71	0.17	0.35	0.11	0.17
21	Public Lands	0.64	0.42	0.51	0.41	0.07	0.13
24	Local Government	0.32	0.68	0.43	0.24	0.40	0.30
	*Macro-average*	0.42	0.54	0.44	0.47	0.26	0.30
	*Micro-average*	0.46	0.57	0.50	0.43	0.25	0.29

Notes: Macro-average is computed with each policy area equally weighted, and micro-average is computed with policy areas weighted by their respective number of bills coded.

**Fig. 8:**
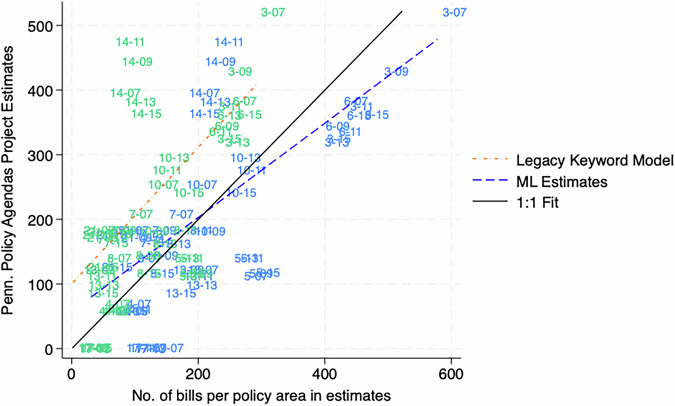
There is a stronger correlation between Machine Learning estimates and the Pennsylvania Policy Database Project than the legacy keyword model. Note: Estimates are labeled with the major topic number (see column 1 of Table 7 and the last two digits of the year.

With those caveats in mind, Fig. 8 shows the machine learning estimates more closely approximate the PPDP coding of the state’s legislative agenda. A “perfect” relationship will align with the dark black line that is a 1:1 relationship. To get into the specifics, Table 7 shows how models compare. The micro-averaged F1 score is 0.50, which is a major improvement upon the legacy dictionary’s micro-averaged F1 score of 0.29. This difference is driven by a dramatic increase in recall, as the machine learning estimate is retrieving about 57 percent of the PPDP bill codes, while the legacy keyword model only recovers a quarter of those estimates. This recall distinction has practical use for researchers. The relatively poor recall of the dictionary model led to the original recommendation to only use those estimates to only make over-time comparisons within a state/policy area, which minimizes the blindspots of the keyword approach^2^. However, the results in Fig. 8 allow researchers to make claims across the agenda such as “there are more bills introduced about transportation in Pennsylvania over the period 2009-2016 than energy,” as researchers can have confidence the relative size of the policy agendas are being evaluated.

In terms of reliability, methods designed to evaluate multi-label classification schemes^26^ show the machine learning estimates perform well. The average ranking loss is 0.11 (0.00 would be perfect, 0.50 would be near chance), which is slightly more ranking loss than the human coder exercise, but that is to be expected because of the measurement error aligning the codebooks. The top-K agreement between the model estimates and PPDP in Table 8 shows at *K* = 3 there is 70 percent agreement. So by considering three guesses per bill, the machine learning method produces a reasonable facsimile of what a human coder can be expected to produce. There also appears to be diminishing returns to the Top-K agreement near 90 percent. This reveals just how difficult this task may be, that even with many guesses it may not be reasonable to generate complete agreement.

**Table 8:** Top-K agreement of model estimates and Pennsylvania codes (n=1,000).

K	Agreement	K	Agreement
1	0.39	6	0.82
2	0.60	7	0.84
3	0.70	8	0.86
4	0.75	9	0.87
5	0.80	10	0.89

## Usage Notes

The data are prepared for applied research. The “individual bill” estimates^23^ dataset has the model predictions for every policy area in case researchers would like to change the *τ* level, and the “master” dataset^24^ indicates each policy area a bill qualifies for with *τ* = 0.50. Each bill has a unique identifier (*dg*_*id*) that aligns with the individual bill estimates and master dataset. The master dataset contains a primary identifier for each bill, which corresponds with the American Joint Operation code (AJO, which is linked to the OpenStates project)^28^. Linking to OpenStates provides more information on the bills, such as more detailed versions of bill legislative histories.

Table 9 explains each component of the AJO key, for example **wv_2011_r1_HB_02801** or West Virginia’s “Creating the ‘Health Care Choice Act’”, which Table 2 showed was assigned to be a **G0300 Health** and **G1510 Insurance** bill. The components of the AJO key are connected with underscores that can be easily split.

**Table 9:** American Joint Operator identification key for each bill.

Component	Description	Example	(notes)
State	Abbreviation	wv	Sample includes the 50 states
Year2	Biennia	2011	This is the first year except for NJ, MS, LA, VA
Session code	regular or special session, number of session	r1	
Prefix	Chamber of origin for bill, bill type	HB	HB is “House Bill”, SB is “Senate Bill States with A for “Assemblies”: CA, NJ, NV, NY, WI. NE’s prefix is L for “Legislature”
Bill Number	Set to five digits	02801	
Postfix	If necessary	n/a	These are only found in: CA, IL, MA, NC, NE, NH, NJ, NV, SC, UT, WI

For cross-sectional research, we suggest aggregating bills by biennia. Most American states have two-year sessions that follow elections taking place in even-numbered years that align with the presidential election cycle (e.g. 2024, 2028, etc.) However, a few states have their elections in odd-numbered years following the presidential cycle. We align the variable tracking each biennium (**Year2**) with the first year of the typical two-year cycle, and the second year for those four states with off-cycle legislative elections (NJ, LA, MS, VA). Another advantage of two-year cycles is that they capture the ebb and flow of an actual legislature. Trying to split a session, like the Massachusetts legislature’s roughly 18 month session, into two separate one-year cycles ignores the fact that many of the bills are introduced in the first few weeks of the session, only to be acted upon in the final few days of the session. The two-year cycle also aligns with each Congress at the national level.

The data can also be aggregated by individual years, using the variable (**Year1**) that shows bills from sessions which began and ended in a single year. This variable is useful for aggregating bills to data that is measured on an annual basis, like lobbying registrations^29^ or Gross State Product.

We also include several of the qualitative descriptors of the bill directly from legiscan as strings, including the session, title of the bill, the year the session started and ended, a qualitative description of the type of bill (e.g. Bill, Resolution, Joint Memorial, etc.) and legiscan’s 5 point status for how far a bill advanced (1: Introduced, 2: Engrossed, 3: Enrolled, 4: Passed, 5: Vetoed). We also provide a url for where the original bill was scraped from the legislature’s website. Some of these links have since become broken, but this provides a useful track to the archival material.

Legiscan provides the partisanship of bill sponsors, we summarize this in a single variable to delineate the primary sponsor (0 = unknown, 1 = Democrats, 2 = Republicans). This is straightforward for states that have only a primary sponsor, we can observe this with Legiscan’s personnel files, but for states that allow multiple sponsors on a bill we take the party of the median primary sponsor.

## Data Availability

Replication code used to generate the policy estimates in python, conduct the analysis and reproduce the figures from this article is hosted by the Open Science Foundation at https://osf.io/xv4w7. In addition to the individual^23^ and master^24^ datasets and replication code, there are also two “readme.txt” files to aid researchers in downloading and using these data.
